# An Adaptive IMU–Visual Multimodal Fusion System for Real-Time Exercise Recognition and Movement Quality Assessment

**DOI:** 10.3390/s26185838

**Published:** 2026-09-15

**Authors:** Zhaoyang Gu, Ruopeng Yang, Yongqi Shi, Dongxu Dai, Chaoyang Li, Bo Huang, Kaige Jiao, Yu Tao, Yongqi Wen, Yihao Zhong, Chen He

**Affiliations:** 1 Graduate School, National University of Defense Technology, Changsha 410073, China; guzhaoyang21@nudt.edu.cn (Z.G.); shiyongqi17@nudt.edu.cn (Y.S.); lichaoyang26@nudt.edu.cn (C.L.); jiaokaige@nudt.edu.cn (K.J.); wenyongqi20@nudt.edu.cn (Y.W.); zhongyihao@nudt.edu.cn (Y.Z.); hechen25@nudt.edu.cn (C.H.); 2 Department of Information and Communication Command, PLA Information Support Force Engineering University, Wuhan 430000, China; daidongxu26@isfeu.edu.cn (D.D.); huangbo19@nudt.edu.cn (B.H.); taoyu22@nudt.edu.cn (Y.T.)

**Keywords:** multimodal fusion, inertial measurement unit, vision-based pose estimation, exercise recognition, movement quality assessment, real-time corrective feedback

## Abstract

Exercise recognition and movement quality assessment remain challenging in supervised exercise training, particularly under viewpoint changes and self-occlusion. Vision-based methods provide spatial posture information but are susceptible to keypoint loss, whereas inertial sensing is less affected by occlusion but provides limited information about global posture geometry. This study presents a dual-stream prototype that combines a nine-axis inertial measurement unit (IMU) with vision-based pose estimation. A 1DCNN-LSTM branch models inertial dynamics, a custom keypoint temporal branch models normalized pose sequences, and a confidence-gated rule adjusts their contributions according to visual keypoint reliability. Owing to the absence of a public synchronized multi-view IMU–vision exercise dataset with the required protocol, we constructed IMV-Exercise, comprising 10 participants, three exercises, and 900 repetition-level samples with side-, front-, and posterior-view recordings. The system achieved 96.0% exercise recognition accuracy under leave-one-subject-out cross-validation. In a separate viewpoint-specific evaluation, the fused output achieved 91.2% action-window accuracy under posterior viewing. Across 50 online trials, the reported recognition accuracy was 96.0%, the mean end-to-end latency was 195 ms, and the recorded maximum was below 210 ms. These results establish feasibility within the studied cohort and exercises; broader generalization and feedback effectiveness require larger, independently controlled evaluations.

## 1. Introduction

In structured or supervised exercise training settings, participants repeatedly perform standardized movements, and coaches or instructors are expected to monitor movement quality and provide timely corrective feedback. During high-frequency or prolonged training, direct observation alone may not provide consistent and quantitative assessments of every repetition, particularly with respect to movement range, trunk stability, joint compensation, and exercise rhythm. Wearable sensors have consequently been investigated for real-time kinematic monitoring and training support across sports and exercise applications [1,2,3,4,5,6,7,8,9]. Such settings allow a lightweight wearable IMU to be fitted before a training session and used together with a camera, making the joint deployment of inertial and visual sensing feasible. Accordingly, the intended application of this study is structured training assistance, in which wearable inertial sensing and vision-based pose estimation are jointly employed for exercise recognition and movement quality assessment.

Existing methods for exercise recognition and movement quality assessment primarily rely on either vision-based or wearable inertial sensor approaches. Vision-based approaches typically use RGB cameras, depth sensors, or human pose estimation algorithms to capture keypoints and then apply spatiotemporal graph models, convolutional networks, Transformers, or other deep learning architectures for exercise recognition [10,11,12,13,14,15,16]. These methods provide rich spatial posture information, making them suitable for analyzing joint angles, limb trajectories, and overall posture deviations [13,14,15]. However, their performance is sensitive to camera viewpoint, lighting variations, background clutter, and limb self-occlusion [10,12,17]. In exercises such as push-ups, sit-ups, and squats, when participants adopt low-body positions, are viewed from the posterior, or experience trunk occlusion, keypoints such as the shoulders, elbows, wrists, hips, and knees may be partially or entirely undetected, reducing the reliability of exercise recognition and posture assessment.

Compared with vision-based approaches, wearable inertial-sensor-based methods do not depend on the camera viewpoint, and offer advantages such as low power consumption, ease of deployment, and greater robustness to occlusion [9,18,19,20,21,22,23,24,25,26,27,28]. Inertial measurement units (IMUs) can collect time-series data, including acceleration, angular velocity, and attitude angles, which are suitable for characterizing movement rhythm, acceleration peaks, rotational states, and periodic dynamic patterns. Traditional inertial-sensor-based recognition methods usually rely on manually designed time-domain or frequency-domain features combined with classifiers such as support vector machines and hidden Markov models [29]. Recent inertial-sensing approaches include model-based joint-motion estimation and deep temporal models for learning movement representations from inertial sequences [5,30,31,32,33,34,35,36,37,38]. However, single-node or sparse inertial sensors mainly capture local dynamic changes and provide limited global spatial posture constraints. As a result, they remain limited in detecting fine-grained posture deviations, such as insufficient elbow flexion, trunk inclination, and knee valgus.

The analysis above indicates that the visual and inertial modalities provide complementary information in real training scenarios. The visual modality offers spatial posture information but is less reliable under occlusion or varying viewpoints. In contrast, inertial sensors provide continuous dynamic information but offer limited insight into global posture relationships. Prior work on multimodal and multi-sensor fusion, including combinations of video and inertial sensing, illustrates the value of complementary measurements [39,40,41,42,43,44,45,46,47,48]. Nevertheless, real-time multimodal exercise systems still face three primary challenges. First, the sampling frequencies and data structures of different modalities differ, requiring temporal alignment. Second, multimodal models incur additional computational cost, requiring a balance between recognition performance and responsiveness. Third, a training assistance system must quantify movement completion, posture deviations, and rhythm stability in addition to classifying the exercise.

To address these challenges, this study presents a dual-stream movement quality assessment prototype that combines a nine-axis IMU with vision-based pose estimation. The system synchronizes IMU signals and visual keypoint sequences through timestamp-based alignment and linear interpolation, and uses pre-extracted human keypoint sequences instead of raw RGB tensors as the temporal visual model input. Metrics based on Dynamic Time Warping (DTW) and Euler-angle stability quantify movement completion, posture deviation, and rhythm variation. A confidence-gated fusion rule then adjusts modality weights according to mean visual keypoint confidence and generates an exercise class together with rule-based corrective cues.

The main contributions of this study are summarized as follows:

1. A confidence-gated IMU–visual fusion pipeline is developed for exercise recognition and posture assessment. It combines temporal information from nine-axis IMU signals with spatial information from visual keypoint sequences and changes the visual contribution according to mean keypoint confidence.
2. Movement quality assessment metrics based on Dynamic Time Warping (DTW) and Euler-angle stability are introduced to quantify movement completion, posture deviation, and rhythm variation.
3. The IMU–Vision Multi-View Exercise Dataset (IMV-Exercise), a self-collected IMU–visual multimodal exercise dataset, is constructed to support the development and evaluation of the proposed system.
4. A real-time training assistance prototype is implemented and evaluated for exercise recognition, viewpoint-dependent visual failure, and aggregate end-to-end latency within the three-exercise IMV-Exercise protocol.

The remainder of this manuscript is organized as follows. Section 2 describes the system architecture, hardware platform, data acquisition protocols, inertial and visual data processing methods, dual-stream models, multimodal fusion strategy, and movement-quality outputs. Section 3 presents the experimental settings, recognition outcomes, baseline comparisons, viewpoint-dependent visual failure analysis, and aggregate online latency measurements. Section 4 interprets the results and addresses limitations, and Section 5 summarizes the key findings. Appendix A reports a separate inertial-only reproducibility check on the public MEx dataset.

## 2. Materials and Methods

This section describes the system architecture, data acquisition protocol, signal preprocessing, model implementation, multimodal fusion strategy, feedback mechanism, and evaluation metrics. The description reports the implementation details that can be verified from the retained experimental specification. General theoretical derivations of established methods, such as MEMS sensing, 1DCNN, LSTM, and DTW, are omitted.

### 2.1. System Overview

**Figure 1:**
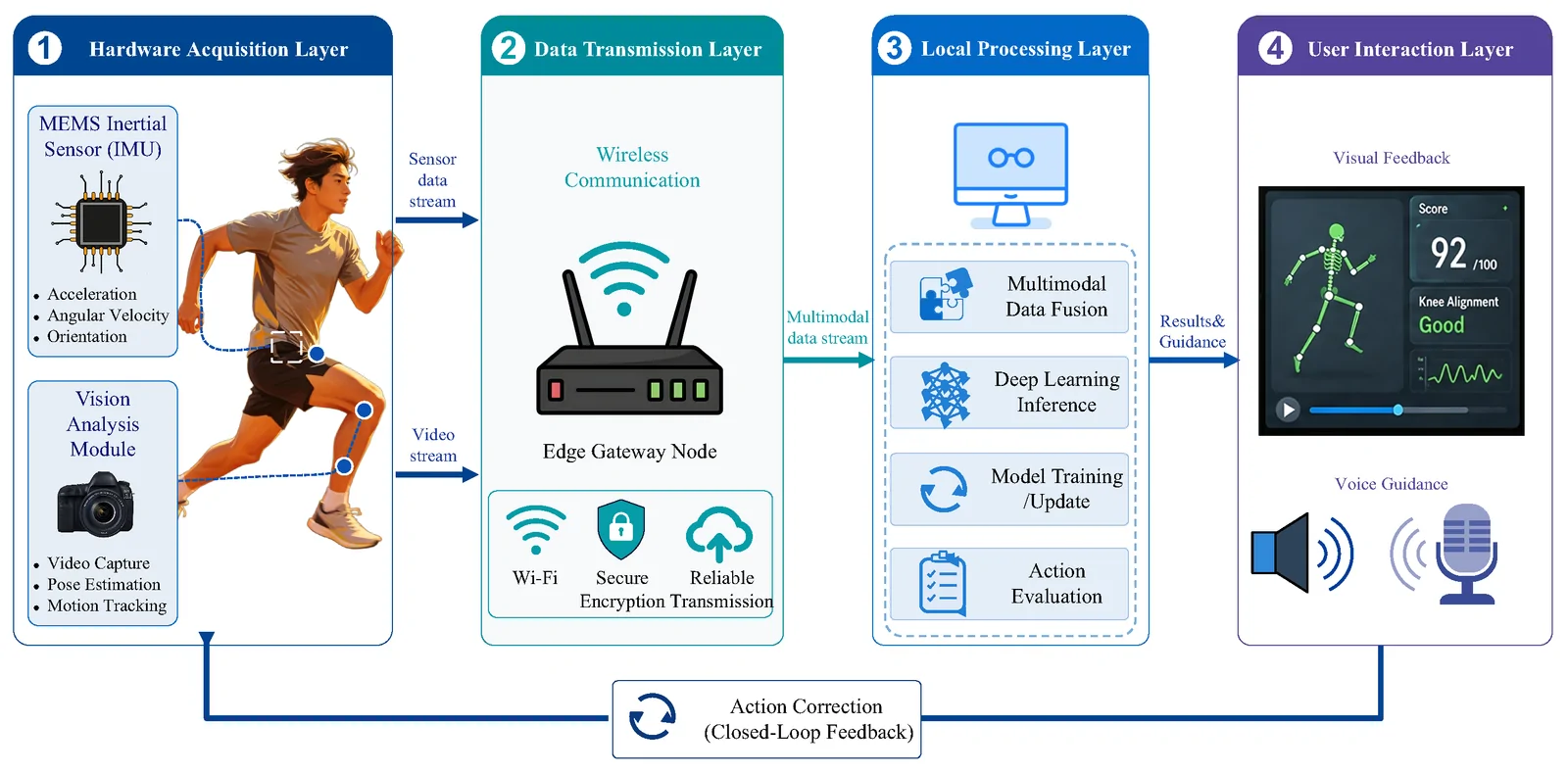
Overall system architecture, comprising the hardware acquisition layer, the data transmission layer, the local processing layer (the IMU and visual branches with confidence-based fusion), and the user interaction layer. The small interface graphic is illustrative; quantitative results are reported separately in the results tables.

The overall system workflow is as follows. First, the JY901 nine-axis IMU (WitMotion, Shenzhen, China) collects inertial data, which are transmitted to a local workstation via an ESP8266-based WiFi module (ESP8266 chip: Espressif Systems, Shanghai, China). Simultaneously, an RGB camera captures the video stream, and human keypoints are extracted on the processing unit using MediaPipe Pose [49,50]. The system then preprocesses the IMU data and keypoint sequences and aligns them using timestamps recorded on the processing unit. The IMU branch employs a 1DCNN-LSTM model, while a custom temporal encoder operating on keypoint sequences forms the visual branch. A confidence-gated rule combines the two branch representations and supplies the exercise class to the movement-quality and feedback modules. The overall system architecture is illustrated in Figure 1.

The IMU input vector at sampling time *t* is defined as

(1) $$x_{imu}\left(t\right)={\left[a_{x}\left(t\right),a_{y}\left(t\right),a_{z}\left(t\right),\omega_{x}\left(t\right),\omega_{y}\left(t\right),\omega_{z}\left(t\right),\phi \left(t\right),\theta \left(t\right),\psi \left(t\right)\right]}^{T},$$

where $a_{x},a_{y},a_{z}$ denote the three-axis acceleration, $\omega_{x},\omega_{y},\omega_{z}$ denote the three-axis angular velocity, and ϕ,θ,ψ denote the roll, pitch, and yaw angles, respectively. The visual input consists of human keypoints estimated by MediaPipe Pose. The visual input at frame *t* is defined as

(2) $$X_{vis}\left(t\right)=\left\{k_{1}\left(t\right),k_{2}\left(t\right),\ldots ,k_{J}\left(t\right)\right\},\;J=17,$$

where $k_{j}\left(t\right)$ represents the normalized coordinates and detection confidence of the *j*-th core keypoint.

The exercise class output is defined as

(3) $$\hat{y}\in \left\{\text{push-up},\text{sit-up},\mathrm{squat}\right\},$$

and the corresponding posterior probability vector is given by

(4) $$p=[p_{push},p_{sit},p_{squat}].$$

The movement quality outputs include movement completion, posture stability, and rhythm deviation. The corrective feedback labels include “good completion”, “insufficient depth”, “torso deviation”, “knee valgus”, and “tempo too fast”.

### 2.2. Hardware Platform and Data Acquisition

**Figure 2:**
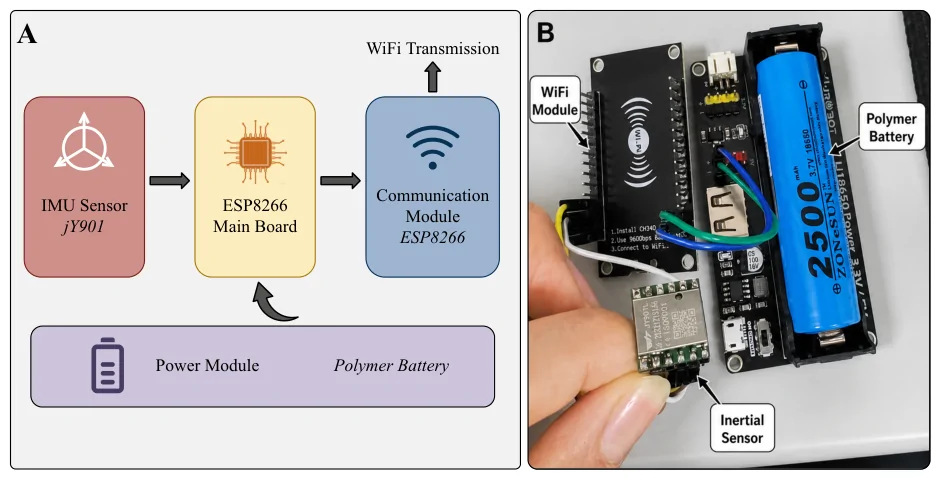
Hardware platform of the wearable sensor node. (**A**) Block diagram showing the JY901 nine-axis IMU, the ESP8266 WiFi module, and the 3.7 V 2500 mAh lithium-polymer battery; (**B**) photograph of the assembled prototype.

The hardware platform, illustrated in Figure 2, consists of a JY901 nine-axis MEMS inertial sensor (WitMotion, Shenzhen, China), an ESP8266 WiFi communication module, a 3.7 V 2500 mAh lithium-polymer battery, a 1920 × 1080 RGB camera, and a local workstation. In the experimental configuration, the JY901 stream was acquired at 200 Hz and provided three-axis acceleration, three-axis angular velocity, and roll–pitch–yaw angles. The protocol conversion ranges were ±16g for acceleration and $\pm 2000^{\circ }/s$ for angular velocity; roll and yaw were represented over $\pm 180^{\circ }$ and pitch over $\pm 90^{\circ }$. The RGB stream was recorded at 30 FPS. These values describe the configuration used in this study rather than a performance validation of the sensor itself.

The ESP8266 module was used only for wireless transmission of inertial data and did not perform deep learning inference. The inertial data were transmitted to the processing unit via the TCP protocol, and CRC16 checksums were used to detect packet errors. Packets that failed checksum validation were excluded from the subsequent model input buffer. A data buffer queue was implemented to temporarily store inertial data during short-term network fluctuations, with a maximum capacity of 500 sampling records. Model inference, cross-modal fusion, and feedback decision-making were all performed on the local workstation.

### 2.3. Participants and Experimental Protocol

Given the limited availability of public synchronized multi-view IMU–vision exercise datasets for supervised exercise training, this study constructed the IMU–Vision Multi-View Exercise Dataset (IMV-Exercise). A total of 10 participants were recruited for data collection, including 6 males and 4 females. Participant age, height, body weight, and the number of exercise samples are summarized in Table 1. IMV-Exercise included three exercise types: push-ups, sit-ups, and squats. Each participant performed 30 standardized repetitions for each exercise type, resulting in 90 exercise samples per participant. In total, IMV-Exercise contained 900 exercise samples.

Model generalization performance was evaluated using leave-one-subject-out (LOSO) cross-validation. In each iteration, data from nine participants were used for training, and the remaining participant’s data served as an independent test set. This process was repeated for all ten participants. This validation scheme prevents data from the same participant from appearing in both the training and test sets, thereby reducing the risk of data leakage and providing an assessment of the model’s ability to generalize to unseen participants.

To evaluate the recognition capability of the visual modality across different viewpoints in IMV-Exercise, exercise videos were collected from side, front, and posterior views. For each exercise–viewpoint pair, 1000 frames were sampled from the recorded viewpoint-specific videos after excluding transition frames before and after exercise execution. These frames were then processed frame by frame to count correctly recognized frames, misclassified frames, and frames for which no exercise label was produced. The cohort was small and demographically narrow (21.0±1.4 years), and only three standardized periodic exercises were included. Consequently, LOSO evaluation measures participant-independent performance within this sampled population; it does not establish generalization to other age groups, clinical populations, exercise types, or unconstrained environments.

### 2.4. IMU Data Preprocessing and Segmentation

The raw IMU data consist of three-axis acceleration, three-axis angular velocity, and three-axis Euler angles. Due to differences in scale and range among channels, as well as potential disturbances from sensor movement and transient noise during acquisition, the system first performs smoothing, differential feature extraction, standardization, and action segmentation on the raw signals.

To reduce the impact of high-frequency noise on action boundary detection, a moving average filter is first applied to the raw sequence:

(5) $$y\left[n\right]=\frac{1}{N}\sum_{k=0}^{N-1}x\left[n-k\right],$$

where x[n] and y[n] represent the discrete signals before and after filtering, respectively, and *N* is the filter window length. Subsequently, the system computes multi-axis differential energy to quantify short-term motion changes. The differential step is set to τ=3, and the differential energy is defined as

(6) $$\Delta d\left[n\right]=\sum_{m\in \{x,y,z\}}\left|s_{m}\left[n\right]-s_{m}\left[n-\tau \right]\right|,$$

where $s_{m}\left[n\right]$ denotes the signal along axis *m* at frame *n*. For acceleration, angular velocity, and angle signals, the system calculates the corresponding three-axis differential energy to enhance dynamic changes at the start and end of each movement.

The segmented action windows are then standardized using Z-score normalization:

(7) $$z_{i}=\frac{x_{i}-\mu }{\sigma },$$

where $x_{i}$ is the *i*-th sample of a given channel, μ and σ are the mean and standard deviation of the channel within the current action segment, and $z_{i}$ is the normalized output. This processing reduces the influence of differing physical units and channel amplitude ranges on model training.

**Figure 3:**
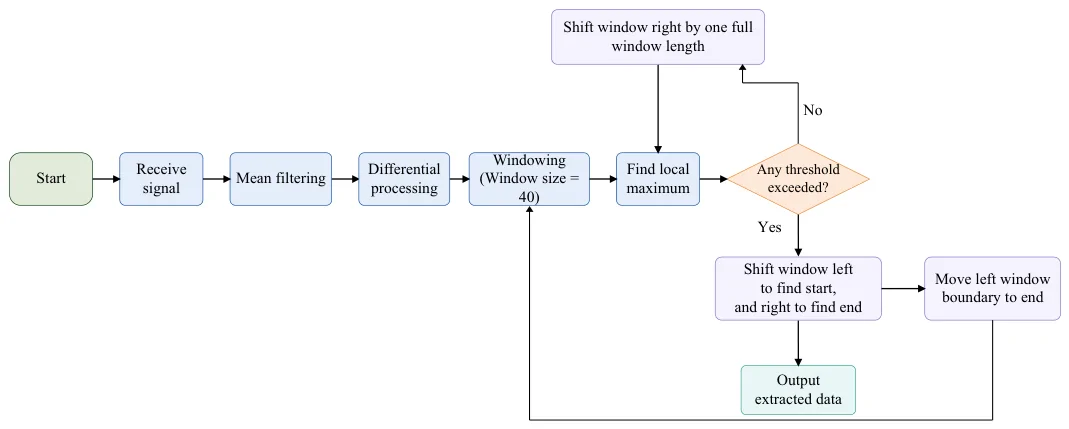
IMU action segmentation workflow based on moving-average filtering, differential feature extraction, and sliding-window threshold detection.

For the continuous IMU data stream, movement segmentation was performed using a sliding window combined with a multi-threshold decision scheme. The sliding-window length was set to 40 frames with a step size of 20 frames. Based on sample statistics and sensor characteristics, the differential thresholds were set as follows: $0.8\;m/s^{2}$ for acceleration, $160^{\circ }/s$ for angular velocity, and $50^{\circ }$ for angles. When any signal within the window exceeded its corresponding threshold, a candidate action segment was triggered. The segment start was determined by searching 20 frames to the left of the window center, and the end was determined by searching 20 frames to the right. After segmenting an action, the left boundary of the sliding window was updated to the current action endpoint to continue scanning the subsequent data stream. If no threshold was exceeded within the window, the entire window shifted 40 frames forward. The action segmentation workflow is illustrated in Figure 3.

The rule-based detector was used to initiate inference during continuous online operation. By contrast, the offline LOSO evaluation used the 900 collected repetition-level samples after action-window construction; therefore, the reported 96.0% recognition accuracy is a classification metric and should not be interpreted as an end-to-end segmentation score. Independent frame-level onset and offset annotations were not retained for IMV-Exercise, so segmentation precision, recall, and boundary-error tolerance cannot be computed from the available records. Boundary shifts may include transition samples or truncate movement phases, and zero padding standardizes input length but does not remove this source of error. The absence of an independent segmentation evaluation is addressed as a limitation in Section 4.

### 2.5. Vision-Based Pose Estimation

The visual modality supplements the spatial posture information that is not captured by the IMU signals. Human keypoints were extracted from RGB video frames using MediaPipe Pose, which initially outputs 33 keypoints. Considering that the exercises in this study primarily involve upper-limb support, trunk flexion, and lower-limb squats, 17 core keypoints—including the shoulders, elbows, wrists, hips, knees, and ankles, together with additional head and foot reference points—were selected from the original set for subsequent temporal modeling in the visual branch.

For each image frame, the image coordinates and confidence score of the *j*-th keypoint are defined as

(8) $$k_{j}\left(t\right)={\left[u_{j}\left(t\right),v_{j}\left(t\right),c_{j}\left(t\right)\right]}^{T},$$

where $u_{j}\left(t\right)$ and $v_{j}\left(t\right)$ denote the normalized two-dimensional image coordinates, and $c_{j}\left(t\right)$ denotes the keypoint detection confidence provided by MediaPipe Pose. To reduce the influence of participant body size and camera distance on the posture sequence, the keypoint coordinates were normalized using the hip center as the local coordinate origin:

(9) $${\tilde{p}}_{j}\left(t\right)=p_{j}\left(t\right)-p_{hip}\left(t\right),$$

where $p_{j}\left(t\right)={\left[u_{j}\left(t\right),v_{j}\left(t\right)\right]}^{T}$ represents the two-dimensional coordinates of the *j*-th keypoint, and $p_{hip}\left(t\right)$ represents the center coordinate of the left and right hip joints. The normalized keypoint sequence forms the input to the visual branch:

(10) $$X_{vis}\in R^{T_{v}\times 17\times 3},$$

where $T_{v}$ is the number of frames in the visual window. The three entries for each of the 17 selected keypoints are the hip-centered horizontal coordinate, the hip-centered vertical coordinate, and the MediaPipe confidence score.

Because the IMU sampling frequency is 200Hz and the video frame rate is 30FPS, the two modalities have different temporal resolutions. To avoid temporal mismatch caused by direct concatenation, the system aligns the two data streams using timestamps recorded on the processing unit. Let the IMU timestamp set be

(11) $$T_{imu}=\left\{t_{1},t_{2},\ldots ,t_{M}\right\},$$

and the video frame timestamp set be

(12) $$T_{vis}=\left\{\tau_{1},\tau_{2},\ldots ,\tau_{N}\right\}.$$

For each IMU sampling time $t_{i}$, the nearest video frame timestamp $\tau_{\zeta (i)}$ is selected as

(13) $$\zeta \left(i\right)=\arg\min_{j}\left|t_{i}-\tau_{j}\right|.$$

When a visual keypoint sequence with the same temporal resolution as the IMU samples is required, linear interpolation is performed between adjacent video frames. If the normalized coordinates of a keypoint at adjacent video frames $\tau_{k}$ and $\tau_{k+1}$ are $p(\tau_{k})$ and $p(\tau_{k+1})$, respectively, then the interpolated coordinate at $t\in (\tau_{k},\tau_{k+1})$ is given by

(14) $$p^{*}\left(t\right)=p\left(\tau_{k}\right)+\frac{t-\tau_{k}}{\tau_{k+1}-\tau_{k}}\left[p\left(\tau_{k+1}\right)-p\left(\tau_{k}\right)\right].$$

This temporal alignment procedure was used to construct synchronized cross-modal action windows. Linear interpolation only estimates keypoint coordinates between two observed 30 FPS frames; it does not create additional visual evidence. It is a local constant-velocity approximation over an inter-frame interval of approximately 33.3 ms and can be inaccurate during rapid direction changes or when either neighboring keypoint is poorly localized. The interpolated confidence was therefore retained as a reliability cue for fusion, but no claim of sub-frame pose accuracy is made. Subsequent model training and online testing were performed based on the aligned action windows.

### 2.6. 1DCNN-LSTM Branch

The IMU branch employs a 1DCNN-LSTM model to extract temporal dynamic features from the motion data. For each successfully segmented action, the nine-axis IMU data are organized as a multi-channel temporal sequence:

(15) $$X_{imu}\in R^{T\times C},\;C=9,$$

where *T* is the length of the action window and *C* is the number of IMU channels. The nine channels correspond to three-axis acceleration, three-axis angular velocity, and three-axis Euler angles. Action segments of different lengths are zero-padded along the temporal dimension to obtain a uniform sequence length $L_{max}$. The padded IMU input is represented as

(16) $${\tilde{X}}_{imu}\in R^{L_{max}\times C},\;C=9.$$

**Figure 4:**
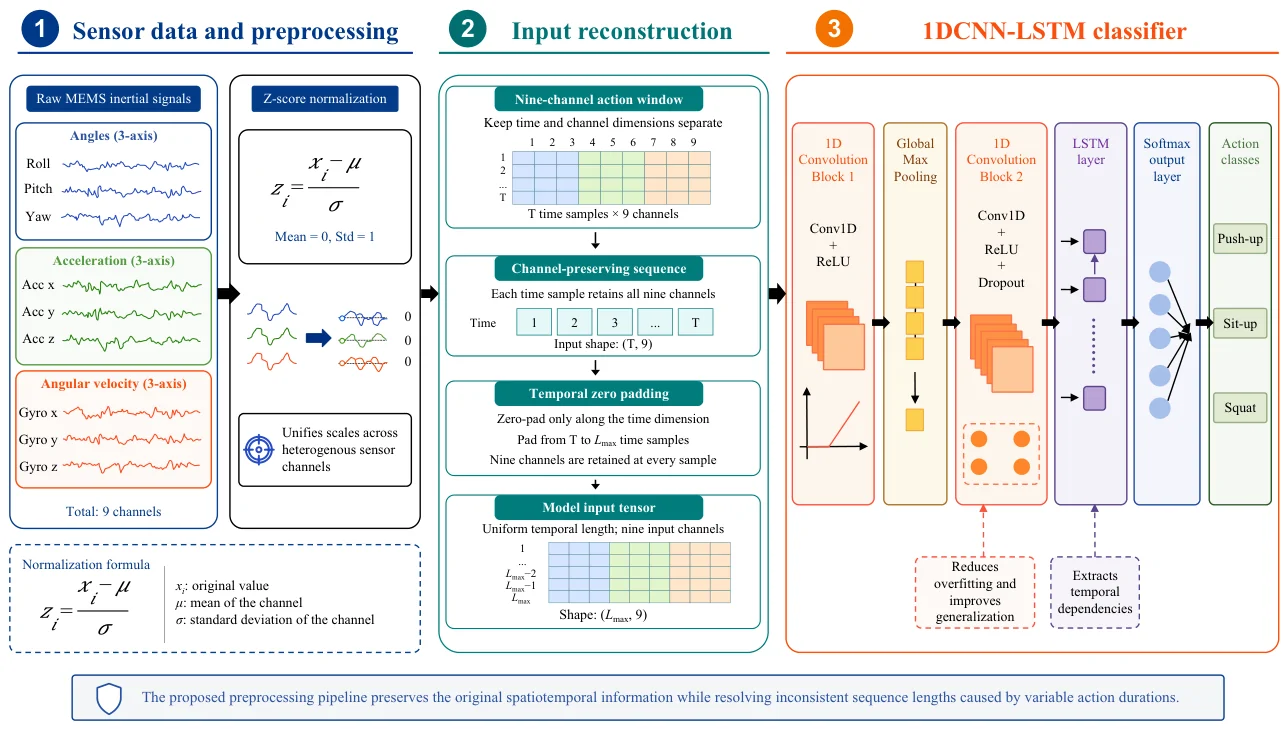
IMU temporal exercise recognition branch based on input reconstruction and 1DCNN-LSTM. Specifically, the proposed preprocessing pipeline preserves the original spatiotemporal information while resolving inconsistent sequence lengths caused by variable action durations. Solid arrows indicate the direction of data processing, while dashed arrows connect explanatory annotations to the corresponding network components. Colors distinguish processing stages and groups of signal channels or network components. Ellipses denote omitted repeated channels, time steps, or network elements.

The IMU branch follows the sequence shown in Figure 4: a first temporal convolution block, global max pooling, a second temporal convolution block with dropout, an LSTM layer, and a three-class Softmax layer. The convolution blocks extract local waveform changes, whereas the LSTM models dependencies among movement phases. The representation supplied to the fusion module is 128-dimensional. The retained experimental specification does not preserve the convolutional kernel widths, filter counts, stride and padding settings, or LSTM hidden dimension. These values are therefore not inferred from a different preliminary implementation, and this reproducibility limitation is stated explicitly in Section 4. The IMU branch outputs posterior probabilities for the three exercise classes:

(17) $$p_{imu}=G_{imu}\left({\tilde{X}}_{imu}\right),$$

where $G_{imu}\left(\cdot \right)$ denotes the 1DCNN-LSTM IMU branch. The input reconstruction and 1DCNN-LSTM branch structure are illustrated in Figure 4.

The model was trained using a multi-class cross-entropy loss function:

(18) $$L_{ce}=-\sum_{k=1}^{K}y_{k}\log\left({\hat{p}}_{k}\right),$$

where K=3 is the number of classes, $y_{k}$ is the one-hot encoded ground-truth label for class *k*, and ${\hat{p}}_{k}$ is the predicted posterior probability for class *k*.

### 2.7. Keypoint Temporal Branch

The visual branch is a custom action-window classifier operating on normalized keypoint sequences. Its use of multiscale temporal representation and local temporal attention was conceptually informed by ActionFormer [51], but the branch is neither the original ActionFormer temporal-localization model nor an implementation of its RGB/video-feature front end. The input has the shape given in Equation (10) and comprises keypoint sequences after hip-center normalization and temporal alignment.

At each frame, the 17 triplets form a 51-dimensional keypoint vector. MediaPipe performs keypoint extraction before this branch, so neither raw RGB tensors nor the original ActionFormer video-feature front end enter the classifier. The retained implementation interface records a 256-dimensional visual representation before fusion and one exercise-class output for each presegmented action window. It does not preserve the fine-grained projection, temporal-convolution, attention-head, or pooling settings. The branch is therefore reported at the verifiable input–output and block level, without claiming exact source-level reproduction of its internal encoder. The visual branch outputs the posterior probabilities of the exercise classes:

(19) $$p_{vis}=G_{vis}\left(X_{vis}\right),$$

where $G_{vis}\left(\cdot \right)$ denotes the adapted keypoint temporal branch.

The visual branch used Focal Loss [52], which down-weights easily classified samples during optimization:

(20) $$L_{focal}=-\alpha_{t}{\left(1-{\hat{p}}_{t}\right)}^{\gamma }\log\left({\hat{p}}_{t}\right),$$

where ${\hat{p}}_{t}$ is the predicted probability corresponding to the ground-truth class, $\alpha_{t}$ is the class weighting factor, and γ is the focusing parameter used to down-weight easily classified samples.

### 2.8. Confidence-Gated Multimodal Fusion

The IMU and visual modalities provide complementary information. The IMU modality does not rely on camera viewpoints and can deliver dynamic information under posterior-view and self-occlusion conditions. The visual modality provides spatial relationships between joints but becomes less reliable when keypoints are occluded or their confidence decreases. The implemented fusion is a low-cost confidence-gated heuristic rather than a learned uncertainty model.

Let $f_{imu}$ denote the high-level features output by the 1DCNN-LSTM IMU branch, and $f_{vis}$ denote the high-level features from the keypoint temporal branch. Before fusion, both modalities are projected to a unified dimensionality via linear mappings:

(21) $${\tilde{f}}_{imu}=f_{imu}W_{imu},$$

(22) $${\tilde{f}}_{vis}=f_{vis}W_{vis},$$

where $W_{imu}$ and $W_{vis}$ are learnable projection matrices. The fused feature is defined as

(23) $$F_{fusion}=w_{imu}{\tilde{f}}_{imu}⊕w_{vis}{\tilde{f}}_{vis},$$

where ⊕ denotes feature concatenation, and $w_{imu}$ and $w_{vis}$ are the fusion weights for the IMU and visual branches, respectively.

The visual confidence $c_{vis}$ is computed as the average detection confidence of the 17 core keypoints within the current action window:

(24) $$c_{vis}=\frac{1}{T_{v}J}\sum_{t=1}^{T_{v}}\sum_{j=1}^{J}c_{j}\left(t\right),$$

where $T_{v}$ is the number of visual frames in the window, J=17 is the number of core keypoints, and $c_{j}\left(t\right)$ is the confidence of the *j*-th keypoint in frame *t*. The fusion weights are determined by

(25) $$\left\{\begin{array}{cc} w_{vis}=c_{vis},\;w_{imu}=1-c_{vis}, & c_{vis}\geq \gamma_{th},\\ w_{vis}=0,\;w_{imu}=1.0, & c_{vis}<\gamma_{th}, \end{array}\right.$$

where $\gamma_{th}=0.45$ is the fixed visual-confidence threshold used in the implementation. When $c_{vis}$ falls below this threshold, the visual contribution is disabled; otherwise, the two weights vary linearly with the mean confidence. All 17 joints contribute equally to $c_{vis}$. Thus, the rule has a discontinuity at $\gamma_{th}$ and does not encode exercise-specific joint importance. The retained evaluation did not include a threshold-sensitivity study, so 0.45 is treated only as the fixed engineering setting used by this prototype, not as an optimized or generally applicable value.

The final fused classification result is given by

(26) $$\hat{y}=G_{fusion}\left(F_{fusion}\right),$$

where $G_{fusion}\left(\cdot \right)$ represents the fusion classification module.

**Figure 5:**
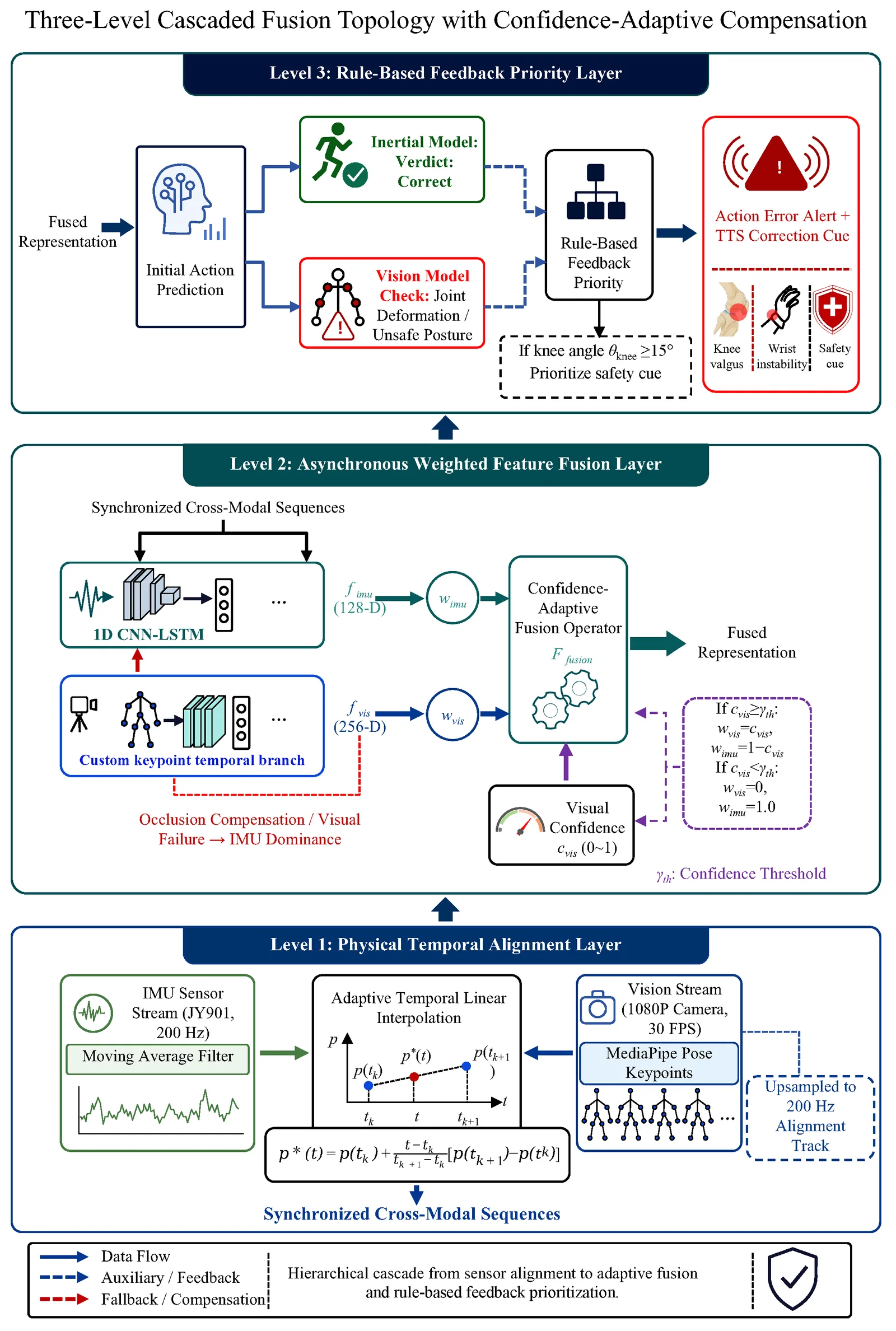
Three-layer cascaded multimodal fusion topology based on visual keypoint confidence. In the schematic, green and teal paths indicate IMU signals and features; blue paths indicate visual signals and features; purple paths indicate confidence control; dark-blue paths connect successive processing layers; and the red dashed path indicates fallback to IMU-dominant processing when visual confidence is insufficient. Dashed auxiliary paths represent feedback or control connections. Ellipses denote repeated feature elements or pose frames omitted for clarity.

The three-layer cascaded multimodal fusion topology is illustrated in Figure 5. The first layer performs cross-modal temporal alignment. The second layer executes feature-level adaptive fusion based on visual keypoint confidence. The third layer generates corrective feedback labels by combining movement quality metrics with predefined feedback rules.

The third layer in Figure 5 is a rule-based feedback-priority module, not a trained “biomechanical logic tree”. It receives the predicted exercise class together with posture and temporal quality flags. If a configured safety flag is present (the prototype includes a squat knee-valgus flag at $\theta_{knee}\geq 15^{\circ }$), the corresponding safety cue has first priority. If no safety flag is present, completion/posture cues derived from the DTW and stability metrics have second priority, followed by rhythm cues. If no threshold is exceeded, no corrective cue is issued. At most one cue is emitted for each action window. These rules generate user-facing labels and are not included in the reported exercise-recognition accuracy.

### 2.9. Real-Time Feedback Strategy

System feedback is determined jointly by the exercise classification results and the movement quality assessment. Once an action segment is segmented, the system first outputs the exercise class along with its posterior probability. Subsequently, corrective feedback labels are generated based on movement completion, posture stability, and rhythm deviation. Feedback labels are categorized into three priority classes: safety, correctness, and rhythm.

Safety-category feedback denotes a predefined posture-rule violation, such as apparent medial knee deviation, pronounced trunk deviation, or unstable wrist support; the label is not a clinical injury-risk assessment. Correctness feedback highlights insufficient movement amplitude, for example, inadequate squat depth, incomplete push-up lowering, or partial sit-up completion. Rhythm feedback addresses timing or speed issues, such as movements performed too quickly, too slowly, or with abnormal pauses between phases. When multiple flags occur simultaneously, the system outputs the highest-priority label according to the order: safety, correctness, and rhythm. Each successfully segmented action window triggers at most one primary feedback label.

Posture stability is characterized by the variation in Euler angles within an action cycle window. Let the action cycle window be *W*, and let the instantaneous posture angle vector be $\Theta \left(t\right)={\left[\phi \left(t\right),\theta \left(t\right),\psi \left(t\right)\right]}^{T}$. The posture stability metric is defined as

(27) $$\sigma_{\Theta }=\sqrt{\frac{1}{\left|W\right|}\sum_{t\in W}{∥\Theta \left(t\right)-\bar{\Theta }∥}_{2}^{2}},$$

where $\bar{\Theta }$ denotes the mean posture angle vector within the window. A larger $\sigma_{\Theta }$ indicates greater trunk or limb posture fluctuation during movement execution.

Movement completion and rhythm deviation are evaluated using Dynamic Time Warping (DTW) [2,53]. Let the feature sequence of the action to be evaluated be $Y=(y_{1},y_{2},\ldots ,y_{M})$, and let the standard action template sequence be $Q=(q_{1},q_{2},\ldots ,q_{N})$. The normalized DTW distance between the two sequences is defined as

(28) $${\bar{D}}_{DTW}\left(Y,Q\right)=\frac{1}{K}\min_{W}\sum_{k=1}^{K}{∥y_{i_{k}}-q_{j_{k}}∥}_{2}^{2},$$

where $W={\left\{\left(i_{k},j_{k}\right)\right\}}_{k=1}^{K}$ is the optimal temporal alignment path between the test sequence and the template sequence, and *K* is the path length. The value of ${\bar{D}}_{DTW}$ is used to quantify the overall difference between the performed movement trajectory and the standard template. If this distance exceeds a predefined threshold, the system identifies the movement as incomplete or deviating from the standard trajectory. Local temporal stretching or compression along the alignment path is further used to detect movements that are too fast, too slow, or contain abnormal pauses between phases.

The feedback results are displayed through a PyQt5-based visualization interface and can also be converted into voice prompts using a text-to-speech (TTS) module. During online operation, the feedback trigger time is recorded for subsequent end-to-end latency analysis.

### 2.10. Evaluation Metrics and Measurement Scope

Exercise recognition performance was evaluated using accuracy, precision, recall, and F1 score. For this three-class classification task, precision, recall, and F1 score were computed for each class and then averaged to obtain the macro metrics. Let $TP_{k}$, $FP_{k}$, and $FN_{k}$ denote the true positives, false positives, and false negatives, respectively, for class *k*, with a total of K=3 classes. The macro-averaged precision, recall, and F1 score are defined as

(29) $$Precision_{macro}=\frac{1}{K}\sum_{k=1}^{K}\frac{TP_{k}}{TP_{k}+FP_{k}},$$

(30) $$Recall_{macro}=\frac{1}{K}\sum_{k=1}^{K}\frac{TP_{k}}{TP_{k}+FN_{k}},$$

(31) $$F1_{macro}=\frac{1}{K}\sum_{k=1}^{K}\frac{2\cdot Precision_{k}\cdot Recall_{k}}{Precision_{k}+Recall_{k}}.$$

Overall accuracy is defined as

(32) $$Accuracy=\frac{N_{correct}}{N_{total}},$$

where $N_{correct}$ is the number of correctly classified samples, and $N_{total}$ is the total number of test samples.

For the visual-modality viewpoint experiment, the numbers of correctly recognized frames, misclassified frames, and unrecognized frames were calculated separately for the side, front, and posterior views using the 1000-frame subsets sampled for each exercise–viewpoint pair. Frame-level accuracy was computed as the proportion of correctly recognized frames among all test frames, with unrecognized frames treated as incorrect predictions. Precision among recognized frames was computed as the proportion of correctly recognized frames among frames that received a valid class prediction. The unrecognized-frame rate was computed as the proportion of frames for which no valid class prediction was produced.

To evaluate whether the system meets the requirements for online training feedback, end-to-end latency was defined as the time interval from the arrival of the last required input frame for the current action window at the processing unit to the generation and triggering of the feedback output. For the *i*-th online inference event, the end-to-end latency is defined as

(33) $$T_{e2e}^{\left(i\right)}=t_{fb}^{\left(i\right)}-t_{in}^{\left(i\right)},$$

where $t_{in}^{\left(i\right)}$ denotes the timestamp at which the last IMU sample and its corresponding video frame for the *i*-th action window arrive at the processing unit, and $t_{fb}^{\left(i\right)}$ denotes the timestamp at which the feedback label is written to the interface or the voice prompt is triggered. The end-to-end latency is further decomposed as

(34) $$T_{e2e}=T_{pre}+T_{align}+T_{infer}+T_{decision}+T_{feedback},$$

where $T_{pre}$ is the time required for data parsing, filtering, and action segmentation; $T_{align}$ is the time required for cross-modal temporal alignment; $T_{infer}$ is the inference time of the 1DCNN-LSTM and keypoint temporal models; $T_{decision}$ is the time required for fusion decision-making and feedback-label generation; and $T_{feedback}$ is the time required for interface updating or voice-prompt triggering. The deployment logger stored only $t_{in}^{\left(i\right)}$ and $t_{fb}^{\left(i\right)}$; it did not retain the five internal timestamps. Consequently, the experiment supports aggregate end-to-end latency only, not a stage-wise latency comparison.

A total of $N_{lat}=50$ online inference events were recorded in the latency test. For each event, $t_{in}^{\left(i\right)}$ and $t_{fb}^{\left(i\right)}$ were recorded, and the average end-to-end latency was calculated as

(35) $${\bar{T}}_{e2e}=\frac{1}{N_{lat}}\sum_{i=1}^{N_{lat}}T_{e2e}^{\left(i\right)},$$

The peak latency was reported as the maximum observed value among the 50 online inference events.

## 3. Results

This section reports the experimental settings and the results obtained on IMV-Exercise. The primary evidence comprises leave-one-subject-out cross-validation, recognition-only baseline comparisons, a frame-level analysis of visual failures across viewpoints, viewpoint-specific fused-output accuracy, and aggregate online deployment measurements. An auxiliary inertial-only reproducibility check on the public MEx dataset is reported separately in Appendix A; it is not treated as an evaluation of the full IMU–visual system.

### 3.1. Experimental Settings

**Implementation Details.** All offline model training and evaluation were conducted using a Python-based deep learning implementation on the local workstation used for system development. The preprocessing, temporal alignment, and action-window construction procedures described in Section 2 were applied to the corresponding inputs used by the IMU branch, visual branch, and fusion model. In each leave-one-subject-out (LOSO) fold, data from one participant were held out exclusively for testing, while data from the remaining nine participants were used for model fitting and validation. No samples from the held-out participant were used for parameter fitting or model selection.

The input and model settings documented in the submitted experimental record are summarized in Table 2. The IMU branch used multi-class cross-entropy, whereas the visual branch used Focal Loss. The baseline HMM, SVM, and ST-GCN values were obtained under the participant-level LOSO protocol using their corresponding input modalities. Because complete baseline configurations and fold-level outputs were not retained, their results are treated as descriptive within-protocol references rather than independently reproduced or controlled component ablations.

**Baseline Algorithms.** Three recognition baselines were included: HMM and SVM using inertial features, and ST-GCN using visual skeleton sequences. The submitted experimental record reports each baseline under the participant-level LOSO protocol. Fold-level baseline predictions and complete baseline configurations needed for independent reproduction were not retained. The comparison therefore concerns descriptive exercise-classification accuracy only; it does not compare movement-quality outputs, model capacity, or online latency and is not used as a controlled ablation.
**Datasets.** The primary evaluation used IMV-Exercise, which contains 10 participants, 900 repetition-level samples, three exercise categories (push-ups, sit-ups, and squats), and side-, front-, and posterior-view recordings. MEx [54] contains 30 subjects performing seven physiotherapy exercises with two body-worn accelerometers, a depth camera, and a pressure mat. As MEx does not reproduce the sensing configuration or outputs of the proposed system, its accelerometer streams were used only for the separate reproducibility check in Appendix A.

### 3.2. Main Results

The fusion system was evaluated on IMV-Exercise using LOSO cross-validation and achieved an overall repetition-level exercise-recognition accuracy of 96.0%. This value measures generalization to held-out participants from the same sampled population and three exercise classes; it should not be extrapolated to other populations or exercises. Table 3 reports recognition accuracy for the fusion system and the three within-protocol reference baselines.

The numerical differences in Table 3 are descriptive. Because the baselines use different sensing inputs and representations, the table does not isolate the contribution of the confidence-gating rule or establish superiority under matched model capacity.

### 3.3. Per-Action and Per-View Performance

To evaluate the recognition performance of the visual modality across different viewpoints, frame-by-frame recognition results were computed for three exercise types under side, front, and posterior views. As described in the experimental protocol, each exercise–viewpoint pair contained 1000 sampled test frames from the corresponding recorded video subset. The retained frame-count summary does not identify each participant’s contribution or the original sampling seed. Moreover, adjacent video frames are correlated observations. The frame counts are therefore used as a descriptive failure analysis and not as an independent-sample statistical comparison. The results are summarized in Table 4.

Based on the frame-by-frame statistics in Table 4, frame-level recognition accuracy was computed by treating unrecognized frames as incorrect predictions. Under the side view, the frame-level accuracies for push-ups, sit-ups, and squats were 100.0%, 95.0%, and 99.2%, respectively. Under the front view, the corresponding accuracies were 93.2%, 88.5%, and 86.6%. Under the posterior view, the accuracies were 0.0%, 25.7%, and 66.2%, respectively. The corresponding micro-averaged accuracies were 98.1%, 89.4%, and 30.6% (Table 5).

**Figure 6:**
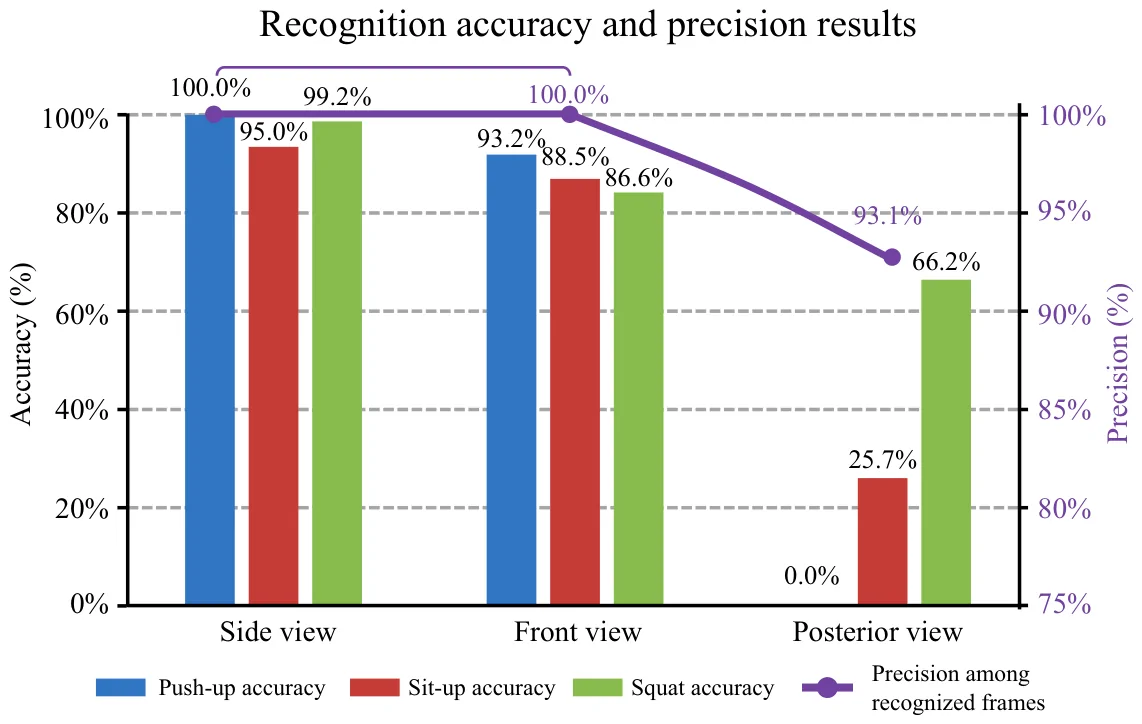
Frame-level recognition accuracy and precision among recognized frames of the visual modality across different viewpoints.

The error pattern was dominated by rejection rather than assignment to another exercise class. Of the 3000 posterior-view frames, 2013 were unrecognized, including all 1000 push-up frames, 675 sit-up frames, and 338 squat frames; only 68 frames were recorded as cross-class errors, all from the sit-up subset. This indicates that posterior self-occlusion mainly caused the pose pipeline to withhold a valid class rather than consistently confuse one exercise pair. The retained aggregate counts do not include the destination label for those 68 frames, so a class-resolved 3×3 confusion matrix cannot be computed from the available records. Figure 6 visualizes accuracy and precision among recognized frames. Rejection rates are reported in Table 5; the aggregate counts are not presented as a confusion matrix.

### 3.4. Viewpoint-Specific Fused Output

The retained fused-decision summary reported accuracies of 100.0%, 95.0%, and 91.2% for the side-, front-, and posterior-view action-window subsets, respectively (Table 6). These action-window values are reported separately from the visual frame-level statistics above. The earlier manuscript placed frame-level visual results and action-window branch results in one table; because those quantities have different evaluation units and the retained IMU-only outputs could not be matched sample by sample to the viewpoint subsets, that comparison was removed. The present results consequently describe the behavior of the fused output across viewpoints but do not constitute a controlled modality ablation.

The lower posterior-view value is consistent with the high visual rejection rate in Table 5. Likely contributing mechanisms include residual errors from partially visible joints, equal weighting of all 17 keypoints in the visual-confidence average, the abrupt gate at $\gamma_{th}=0.45$, and alignment error during rapid movement phases. These mechanisms are plausible failure sources rather than class-resolved causal estimates because per-sample confidence traces and matched single-branch predictions were not retained.

### 3.5. Latency and Deployment Performance

To evaluate online operation, 50 exercise-detection trials were conducted with the complete pipeline running. The reported online recognition accuracy was 96.0%. The mean end-to-end response latency was 195 ms and the recorded maximum was below 210 ms (Table 7). Only aggregate input and feedback timestamps were retained, so dispersion statistics and stage-wise latency cannot be computed from the available log. The baseline algorithms were not integrated into the same acquisition and feedback pipeline; a baseline-latency comparison is therefore not reported.

**Figure 7:**
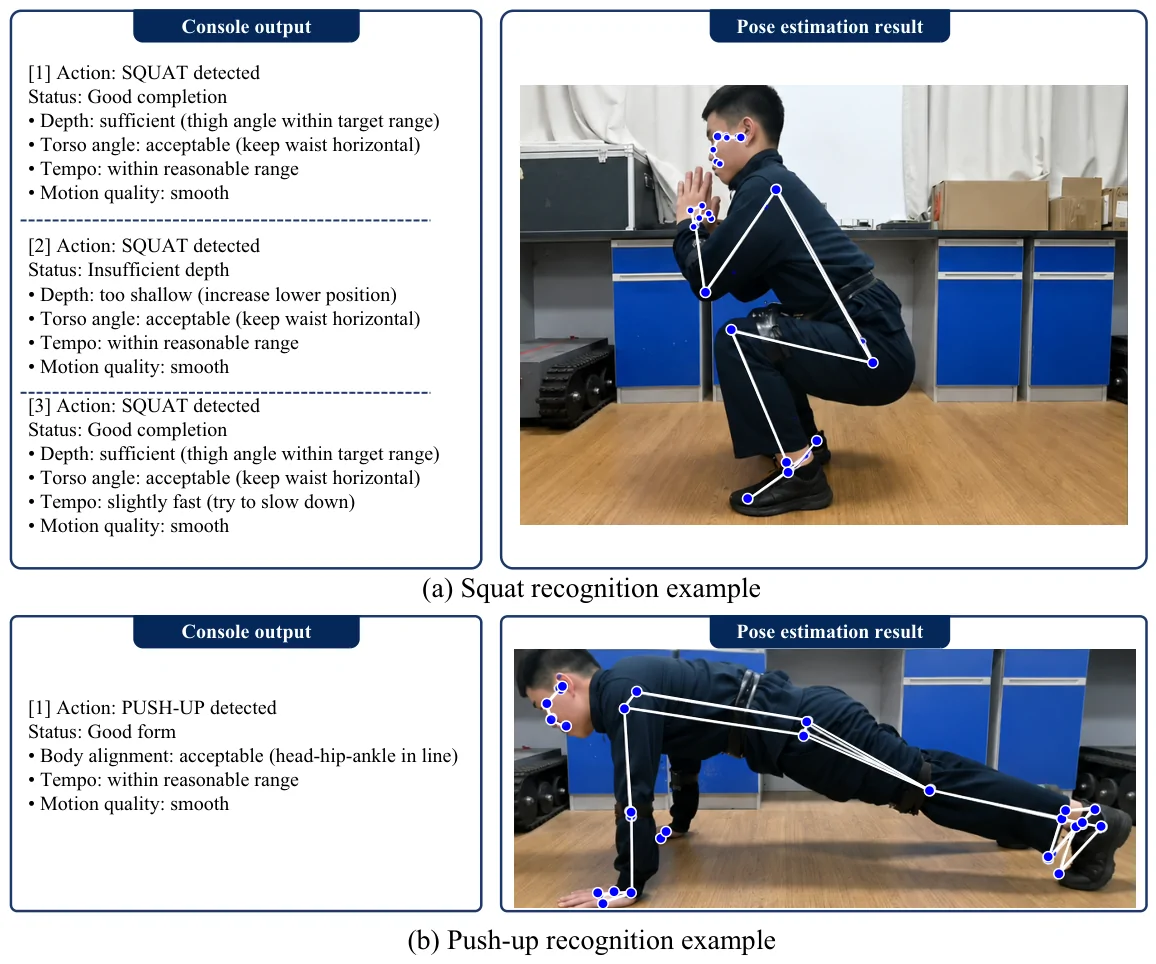
Examples of online system operation: (**a**) squat recognition and (**b**) push-up recognition. In each panel, the console output (left) shows the exercise class and feedback label, while the pose estimation result (right) shows the detected visual keypoints. The bracketed numbers in the console outputs are sequential event indices, not reference citations.

Figure 7a shows an example of online system operation for squat recognition, and Figure 7b shows an example for push-up recognition. In each example, the console output on the left displays the exercise class and feedback label generated by the system, while the pose estimation result on the right shows the detected visual keypoints.

## 4. Discussion

This study developed a dual-stream prototype that integrates a nine-axis IMU with vision-based pose estimation for three periodic exercises. The fusion system achieved 96.0% repetition-level recognition accuracy under LOSO evaluation on IMV-Exercise. In the separate online test, the reported recognition accuracy was 96.0%, with a mean end-to-end latency of 195 ms and a recorded maximum below 210 ms. These results establish feasibility within the sampled young-adult cohort and protocol. They do not by themselves establish broad population generalization, superiority over every matched single-modality model, clinical benefit, or the effectiveness of the generated corrective cues.

### 4.1. Interpretation of the Fusion and Failure Results

The fusion design is motivated by complementary sensing characteristics. IMU signals represent acceleration, angular velocity, and attitude changes without depending on camera visibility, whereas keypoint sequences provide image-plane joint geometry. The present experiment shows that the fused output remained above 90% across the three recorded viewpoints, but it does not isolate how much of that performance is attributable to each branch or to the confidence gate. A controlled modality ablation would require the IMU-only, visual-only, and fused models to be evaluated on the same action windows with the same target labels and metric. Those matched per-sample outputs were not retained, so the earlier cross-unit comparison was removed.

The visual frame analysis provides a clearer failure signal. The unrecognized-frame rate increased from 1.9% in the side view to 67.1% in the posterior view, with all 1000 posterior push-up frames rejected. Only 68 posterior frames were assigned to an incorrect class; the remaining 2013 errors were rejections. Thus, loss of detectable or sufficiently confident keypoints, rather than systematic confusion between two exercise classes, was the dominant recorded visual failure. The fused action-window accuracy also decreased from 100.0% in the side view to 91.2% in the posterior view. Because the frame-level and action-window results have different units, their numerical difference is not an estimate of fusion gain.

The posterior-view decrease also identifies conditions under which the gate can fail. Averaging confidence over all 17 joints can mask the loss of exercise-critical joints, and a window just above the fixed threshold still contributes visual features despite potentially unreliable local geometry. Temporal interpolation and action-boundary offsets can add further mismatch during rapid phases. These explanations are consistent with the implemented pipeline, but they remain hypotheses because joint-level confidence traces and class-resolved fused errors were not preserved.

### 4.2. Robustness Under Occlusion and Viewpoint Changes

Occlusion robustness is a key consideration in the design of the proposed system. Experimental results indicate that the visual modality exhibits a pronounced performance decline under posterior-view conditions, particularly for push-ups and sit-ups, where core keypoints are frequently occluded by the trunk or the ground. This limitation is consistent with the fundamental constraints of visual pose estimation: when key joints such as the shoulders, elbows, wrists, hips, or knees are not visible or have reduced confidence, the visual temporal model cannot reliably capture the complete spatial posture trajectory.

The IMU branch is unaffected by camera visibility and continues to supply motion signals under posterior-view or self-occlusion conditions. The confidence gate was designed to reduce the visual contribution when mean keypoint confidence falls below 0.45. The current data show that the complete pipeline remained operational under posterior viewing, but they do not quantify the counterfactual performance of a continuously weighted gate, an IMU-only fallback, or a learned uncertainty model on the same samples.

The current fusion rule is therefore an engineering heuristic. It gives equal importance to all selected joints, although shoulders, elbows, and wrists are more informative for push-ups and hips, knees, and ankles are more informative for squats. It also changes discontinuously at 0.45, and no threshold-sensitivity analysis was available. Future work should compare this operating point with a prespecified threshold sweep, use exercise-dependent or learned joint weights, calibrate confidence, and replace the hard gate with a smooth transition or uncertainty-aware fusion rule.

### 4.3. Comparison with Baseline Methods

The fusion system had the highest descriptive accuracy in the within-protocol comparison (96.0%), followed by HMM (94.8%), SVM (89.0%), and ST-GCN (76.2%). HMM and SVM used inertial representations, whereas ST-GCN used visual skeleton input. The results therefore compare complete recognition pipelines rather than isolating a single architectural component.

The practical distinction of the prototype is its integration of inertial dynamics, visual posture representation, movement-quality metrics, and a feedback interface in one pipeline. However, the baseline table does not control for input information, parameter count, feature engineering, or computational budget. It cannot establish that the fusion rule itself is responsible for the observed ranking.

Accordingly, the comparison should be interpreted only as a reference within IMV-Exercise and the reported LOSO split. A future controlled study should evaluate matched-capacity single-branch and fusion models on identical action windows, report per-fold uncertainty, and retain per-sample predictions for paired statistical analysis.

### 4.4. Deployability and Real-Time Feedback

The 50 online trials yielded a mean end-to-end latency of 195 ms and a recorded maximum below 210 ms. Under the current hardware and software configuration, the prototype therefore generated its output within the reported sub-second interval after receiving the final input required for an action window. This measurement begins after the action window is available and should not be confused with the duration of the exercise repetition or the unvalidated action-boundary detection delay.

The implementation has three practical characteristics. First, the ESP8266 module transmits IMU data while deep-model inference is performed on the local workstation. Second, the visual branch receives selected keypoint sequences rather than raw RGB tensors. Third, movement-quality metrics are mapped to a limited set of deterministic output labels. These design choices describe the implemented prototype; their individual effects on computation time were not isolated experimentally.

The latency evidence remains limited. The logger retained only the input-arrival and feedback-trigger timestamps, so standard deviation, percentile statistics, and stage-wise times for preprocessing, alignment, keypoint extraction, branch inference, fusion, and interface output cannot be computed from the available records. The 50 trials also came from one implementation environment. Future evaluation should retain every internal timestamp and report the distribution of each stage across devices and operating conditions.

### 4.5. Feedback Scope and Practical Implications

The implemented module maps classification and movement-quality outputs to one prioritized text or speech cue. The present evaluation demonstrates cue generation and records the time required to trigger the output; it does not evaluate whether participants subsequently improve their technique. The earlier exploratory group comparison lacked retrievable group sizes, randomization details, a prespecified assessment scale, rater agreement, confidence intervals, and statistical tests, and has therefore been removed from the Results section. A feedback-effectiveness claim would require a prospectively designed controlled study with transparent allocation, blinded assessment, inter-rater reliability, effect sizes, uncertainty intervals, and a defined primary outcome.

From a practical perspective, the system is better suited as an assistive tool for coaches or trainees rather than a replacement for professional instruction. It provides continuous, quantifiable, and recordable information on exercise recognition and feedback, reducing the burden of manual observation in group training scenarios. However, for complex injury risk assessment, individualized training load adjustments, and medical evaluation, professional judgment that considers the participant’s physical condition remains essential.

Beyond on-site group training, the sensor-and-keypoint pipeline may also support remote or hybrid practice-oriented instruction, in which trainees use accessible sensing devices to transmit movement descriptors and receive automated cues while an instructor reviews summarized performance. Related work has explored smartphones and exergame controllers as bring-your-own-device sources of kinematic data for online sport and exercise science activities, as well as vision-based exercise biofeedback for telerehabilitation [8,55]. This application remains prospective: deployment outside supervised laboratory conditions would require validation across heterogeneous consumer devices, network conditions, home environments, and instructor workflows.

### 4.6. Limitations and Future Work

This study has several limitations. First, the retained final-model specification records the branch order, losses, input and fusion feature dimensions, optimizer, learning rate, batch size, training limit, early-stopping patience, and dropout rate, but not every convolutional kernel, filter count, stride, padding rule, recurrent hidden dimension, or keypoint-encoder attention setting. The method can therefore be assessed at the block and interface level, but exact source-level reproduction of the trained architecture is not possible from the retained record. Future releases should archive executable configurations, source code, model summaries, and fixed checkpoints together with the data-processing scripts.

Second, IMV-Exercise contains only 10 participants with a narrow age distribution and 900 repetitions. Although LOSO separation prevents the held-out participant from entering model fitting, it does not cover broader variation in age, training status, body type, clinical condition, or exercise technique. Future work should include a larger and more diverse cohort and report uncertainty across participants rather than only pooled accuracy.

Third, the current experiments include only three exercise types: push-ups, sit-ups, and squats. These exercises are highly periodic, making them suitable for IMU temporal modeling and visual pose modeling, but they do not represent the full range of training exercises. Movements such as lunges, pull-ups, burpees, weighted exercises, agility drills, or combination exercises exhibit substantial variations in movement speed, joint involvement, and occlusion patterns. Future research should expand the set of exercise types and evaluate the model’s generalization across different exercises, environments, and devices.

Fourth, the current experiments used relatively fixed camera and sensor placements. Lighting, background complexity, multi-person occlusion, camera distance, sensor loosening, orientation changes, and wireless loss were not systematically varied. The viewpoint analysis isolates only three recorded camera directions and should not be treated as evidence for unconstrained deployment.

Continuous acquisition of video-derived pose and wearable-sensor signals also raises privacy and data-protection considerations. Although the current pipeline uses selected keypoints rather than raw RGB tensors for model input, source video and synchronized motion traces can still contain identifying or sensitive behavioral information. Future deployments should therefore implement data minimization, consent specific to data reuse, encryption in transit and at rest, access controls, retention limits, and, where feasible, on-device processing or privacy-preserving motion representations. Visual animation of kinematic data provides one related approach for reducing exposure while retaining analytic utility [56].

Fifth, independent onset and offset annotations were not retained, so segmentation precision, recall, boundary error, and classification robustness to shifted action windows could not be measured. Zero padding makes the network input length uniform but does not correct missing movement phases or included transition samples. Future data collection should preserve frame-level boundaries and evaluate both segmentation metrics and classification under controlled temporal offsets.

Sixth, linear interpolation between 30-FPS visual observations assumes locally constant keypoint velocity. It can be inaccurate during rapid reversals and cannot recover occluded visual evidence. Higher-rate cameras, motion-aware interpolation, or alignment models that propagate uncertainty should be compared in future work.

Seventh, the current fusion rule uses equal joint contributions, a fixed discontinuous threshold, and no reported sensitivity analysis. The retained summaries also do not support a fair matched-window modality ablation or a class-resolved confusion matrix. These analyses require per-sample branch predictions and confidence traces and should be part of a preregistered evaluation protocol.

Eighth, this study evaluates recognition, movement-quality computation, and output latency, but not long-term training benefit or injury incidence. Such outcomes require longitudinal intervention designs and validated exercise-science or clinical endpoints.

In summary, the results support the feasibility of the integrated prototype within IMV-Exercise and the recorded online setting. Stronger claims about fusion superiority, segmentation robustness, feedback effectiveness, population generalization, or injury prevention require the controlled and data-complete evaluations outlined above.

## 5. Conclusions

This study presents a dual-stream prototype that combines nine-axis IMU sensing with vision-based keypoint sequences for exercise recognition and movement-quality computation. On IMV-Exercise, the fusion system achieved 96.0% repetition-level accuracy under LOSO evaluation. In a separate 50-trial online test, the reported recognition accuracy was 96.0%, with a mean end-to-end latency of 195 ms and a recorded maximum below 210 ms. The viewpoint analysis also quantified a substantial increase in visual rejection under posterior viewing, while the fused action-window output remained at 91.2% accuracy.

The evidence is limited to 10 young adults, three periodic exercises, fixed acquisition conditions, aggregate latency logs, and retained summary outputs. It does not establish a controlled modality advantage or feedback effectiveness. Future work should retain executable model specifications, per-sample predictions, and internal timestamps; validate segmentation and threshold sensitivity; use matched-window ablations; and evaluate larger and more diverse cohorts under realistic conditions.

## Figures and Tables

**Table 1:** Participant demographics and IMV-Exercise composition (n=10).

Sex	Number	Age (y)	Height (cm)	Weight (kg)	Exercise Samples
Male	6	21.2±1.5	176.5±5.2	72.3±6.8	540
Female	4	20.8±1.2	164.2±4.5	56.8±5.1	360
Overall	10	21.0±1.4	171.6	66.1	900

Overall height and weight are reported as means only.

**Table 2:** Documented input, model, and evaluation settings.

Item	Setting
Training environment	Python-based deep learning implementation on a local workstation
Evaluation protocol	Ten LOSO folds; one participant held out for testing in each fold
Optimizer and learning rate	Adam with an initial learning rate of $1\times 10^{-3}$
Training schedule	Mini-batch size 32; maximum 50 epochs; early stopping patience 10
Regularization	Dropout rate 0.1 in the trainable deep branches
IMU input	Nine channels sampled at 200 Hz; variable action windows zero-padded to a common length
Visual input	Seventeen hip-centered keypoints, represented by two coordinates and one confidence value at 30 FPS
IMU representation	Two temporal convolution blocks, global max pooling, dropout, LSTM, and three-class Softmax; 128-dimensional fusion feature
Visual representation	51-dimensional keypoint vector per frame; 256-dimensional representation before fusion; one class output per action window
Training objectives	Multi-class cross-entropy for the IMU branch; Focal Loss for the visual branch
Baseline models	HMM, SVM, and ST-GCN reported under the participant-level LOSO protocol

**Table 3:** Exercise-recognition accuracy on IMV-Exercise under the participant-level LOSO protocol.

Algorithm	Accuracy
HMM	94.8%
SVM	89.0%
ST-GCN	76.2%
Fusion system	96.0%

**Table 4:** Frame-by-frame recognition results of the visual modality for different exercises and viewpoints.

Exercise	Viewpoint	Total	Correct	Misclassified	Unrecognized
Push-up	Side	1000	1000	0	0
Push-up	Front	1000	932	0	68
Push-up	Posterior	1000	0	0	1000
Sit-up	Side	1000	950	0	50
Sit-up	Front	1000	885	0	115
Sit-up	Posterior	1000	257	68	675
Squat	Side	1000	992	0	8
Squat	Front	1000	866	0	134
Squat	Posterior	1000	662	0	338

**Table 5:** Visual-modality recognition metrics for different viewpoints.

Metric	Side View	Front View	Posterior View
Push-up frame-level accuracy	100.0%	93.2%	0.0%
Sit-up frame-level accuracy	95.0%	88.5%	25.7%
Squat frame-level accuracy	99.2%	86.6%	66.2%
Micro-averaged frame-level accuracy	98.1%	89.4%	30.6%
Precision among recognized frames	100.0%	100.0%	93.1%
Unrecognized-frame rate	1.9%	10.6%	67.1%

**Table 6:** Viewpoint-specific action-window accuracy of the fused output on IMV-Exercise.

Output	Side View	Front View	Posterior View
Fusion system	100.0%	95.0%	91.2%

**Table 7:** Recognition and latency results during online deployment.

Evaluation Item	Result
Number of online tests	50
Average end-to-end response latency	195ms
Peak end-to-end response latency	<210ms
Online exercise-recognition accuracy	96.0%

## Data Availability

The data presented in this study are available on request from the corresponding author. The data are not publicly available due to privacy restrictions.

## Supplementary Materials

The following supporting information can be downloaded at: https://www.mdpi.com/article/10.3390/s26185838/s1, File S1, Python 3 script used for the auxiliary inertial-only reproducibility check on the public MEx dataset.

## Abbreviations

The following abbreviations are used in this manuscript:

IMU	Inertial Measurement Unit
MEMS	Micro-Electro-Mechanical Systems
CNN	Convolutional Neural Network
1DCNN	One-Dimensional Convolutional Neural Network
LSTM	Long Short-Term Memory
DTW	Dynamic Time Warping
TTS	Text-to-Speech
LOSO	Leave-One-Subject-Out
FPS	Frames Per Second
ST-GCN	Spatial-Temporal Graph Convolutional Network
HMM	Hidden Markov Model
SVM	Support Vector Machine
MEx	Multi-modal Exercise Dataset

## Appendix A. Auxiliary Inertial-Only Reproducibility Check on MEx

The public MEx dataset [54] contains recordings from 30 subjects performing seven physiotherapy exercises. It includes thigh and wrist accelerometers, a depth camera, and a pressure mat. This appendix reports a separate reproducibility check using only the two accelerometer streams. It does not evaluate the visual branch, confidence gate, movement-quality module, or online feedback pipeline used with IMV-Exercise.

Each available exercise trial was processed as one sample. The public release yielded 239 valid trials: 30 trials for each of six exercise classes and 59 trials for the fourth exercise class, which contains a second trial for most subjects. For each accelerometer, deterministic full-sequence features were computed from the three axes and vector magnitude. These features included sample count; distribution and amplitude statistics; first-difference summaries; pairwise axis correlations; and the mean and standard deviation within three equal-duration temporal segments. This procedure produced 96 features per sensor and 192 features when the thigh and wrist feature vectors were concatenated.

Subjects were assigned to five folds by sorted subject identifier, with six subjects held out in each fold and no subject appearing in both training and test data within a fold. Feature standardization was fitted on the training subjects only. A multinomial Softmax classifier was trained for 160 epochs with an initial learning rate of 0.025, epoch-wise decay, an $L_{2}$ coefficient of $5\times 10^{-4}$, and random seed 2026. Table A1 reports the unweighted mean of the five fold-level metrics. The values were reproduced by rerunning the analysis script included as Supplementary File S1.

**Table A1:** Auxiliary inertial-only results on the public MEx dataset under five-fold subject-independent evaluation.

Accelerometer Input	Trials	Accuracy	Macro-F1
Thigh	239	86.6%	85.5%
Wrist	239	63.6%	60.1%
Thigh and wrist	239	89.5%	88.7%

The dual-accelerometer feature set produced the highest mean accuracy in this auxiliary check. Because the task, sensing configuration, feature representation, classifier, and validation protocol differ from those of IMV-Exercise, these values must not be compared numerically with the 96.0% multimodal result. They show only that the accompanying inertial-only analysis is reproducible on a public exercise dataset.

## References

[1] Czekaj Ł., Kowalewski M., Domaszewicz J., Kitłowski R., Szwoch M., Duch W. (2024). Real-time sensor-based human activity recognition for eFitness and eHealth platforms. Sensors.

[2] Seo N.J., Coupland K., Finetto C., Scronce G. (2024). Wearable sensor to monitor quality of upper limb task practice for stroke survivors at home. Sensors.

[3] Prabhu G., O’Connor N.E., Moran K. (2020). Recognition and repetition counting for local muscular endurance exercises in exercise-based rehabilitation: A comparative study using artificial intelligence models. Sensors.

[4] Bang G.-S., Park S.-B. (2024). Workout classification using a convolutional neural network in ensemble learning. Sensors.

[5] Radhakrishnan M., Premkumar V.J., Prahaladhan V.B., Mukesh B., Nithish P. (2024). Convolution neural network based multi-class classification of rehabilitation exercises for diastasis recti abdominis using wearable EMG-IMU sensors. Eng. Comput..

[6] Lee S., Lim Y., Lim K. (2024). Multimodal sensor fusion models for real-time exercise repetition counting with IMU sensors and respiration data. Inf. Fusion.

[7] Spilz A., Munz M. (2023). Automatic assessment of functional movement screening exercises with deep learning architectures. Sensors.

[8] Barzegar Khanghah A., Fernie G., Roshan Fekr A. (2023). Design and validation of vision-based exercise biofeedback for tele-rehabilitation. Sensors.

[9] Rana M., Mittal V. (2021). Wearable sensors for real-time kinematics analysis in sports: A review. IEEE Sens. J..

[10] Iosifidis A., Tefas A., Pitas I. Neural representation and learning for multi-view human action recognition. Proceedings of the 2012 International Joint Conference on Neural Networks (IJCNN).

[11] Shi L., Zhang Y., Cheng J., Lu H. (2020). Skeleton-based action recognition with multi-stream adaptive graph convolutional networks. IEEE Trans. Image Process..

[12] Yan S., Xiong Y., Lin D. Spatial temporal graph convolutional networks for skeleton-based action recognition. Proceedings of the Thirty-Second AAAI Conference on Artificial Intelligence (AAAI).

[13] Liu Y., Qiu C., Zhang Z. (2024). Deep learning for 3D human pose estimation and mesh recovery: A survey. Neurocomputing.

[14] Kappan M.M., Sandoval E.B., Meijering E., Cruz F. (2026). A survey on deep learning for 2D and 3D human pose estimation. Artif. Intell. Rev..

[15] Ji H., Wang L., Zhang Y., Li Z., Wei C. (2024). A review of human pose estimation methods in markerless motion capture. Comput.-Aided Des. Appl..

[16] Zakir A., Salman S.A., Takahashi H. (2024). SOCA-PRNet: Spatially oriented attention-infused structured-feature-enabled PoseResNet for 2D human pose estimation. Sensors.

[17] Aguilar-Ortega R., Berral-Soler R., Jiménez-Velasco I., Romero-Ramírez F.J., García-Marín M., Zafra-Palma J., Muñoz-Salinas R., Medina-Carnicer R., Marín-Jiménez M.J. (2023). UCO Physical Rehabilitation: New dataset and study of human pose estimation methods on physical rehabilitation exercises. Sensors.

[18] Caprioli L., Romagnoli C., Della Loggia L., Najlaoui A., Campoli F., Cariati I., Edriss S., Bonanni R., Paté A., Chadefaux D. (2026). The use of inertial sensors in sports: A bibliometric network and cluster analysis. Smart Health.

[19] Xia K., Huang J., Wang H. (2020). LSTM-CNN architecture for human activity recognition. IEEE Access.

[20] Gao W., Zhang L., Teng Q., He J., Wu H. (2021). DanHAR: Dual attention network for multimodal human activity recognition using wearable sensors. Appl. Soft Comput..

[21] Kwon H., Abowd G.D., Plötz T. (2021). Complex deep neural networks from large scale virtual IMU data for effective human activity recognition using wearables. Sensors.

[22] Khan Z.N., Ahmad J. (2021). Attention induced multi-head convolutional neural network for human activity recognition. Appl. Soft Comput..

[23] Tang Y., Zhang L., Min F., He J. (2023). Multiscale deep feature learning for human activity recognition using wearable sensors. IEEE Trans. Ind. Electron..

[24] McGrath T., Stirling L. (2020). Body-worn IMU human skeletal pose estimation using a factor graph-based optimization framework. Sensors.

[25] Nouriani A., McGovern R.A., Rajamani R. (2024). Vector-based inertial poser: Human pose estimation with high gain observer and deep learning using sparse IMU sensors. Biomed. Signal Process. Control.

[26] Kim M., Lee S. (2022). Fusion poser: 3D human pose estimation using sparse IMUs and head trackers in real time. Sensors.

[27] Yi X., Zhou Y., Xu F. (2021). TransPose: Real-time 3D human translation and pose estimation with six inertial sensors. ACM Trans. Graph..

[28] Liang J.Y., Chou L.-S. (2024). Center of mass acceleration during walking: Comparison between IMU and camera-based motion capture methodologies. Wearable Technol..

[29] Mannini A., Sabatini A.M. (2010). Machine learning methods for classifying human physical activity from on-body accelerometers. Sensors.

[30] Dirgová Luptáková I., Kubovčík M., Pospíchal J. (2022). Wearable sensor-based human activity recognition with transformer model. Sensors.

[31] Hochreiter S., Schmidhuber J. (1997). Long short-term memory. Neural Comput..

[32] Seenath S., Dharmaraj M. (2023). Conformer-based human activity recognition using inertial measurement units. Sensors.

[33] Hoang T., Shiao Y. (2023). New method for reduced-number IMU estimation in observing human joint motion. Sensors.

[34] Zheng Z., Ma H., Yan W., Liu H., Yang Z. (2021). Training data selection and optimal sensor placement for deep-learning-based sparse inertial sensor human posture reconstruction. Entropy.

[35] Puchert P., Ropinski T. (2023). A3GC-IP: Attention-oriented adjacency adaptive recurrent graph convolutions for human pose estimation from sparse inertial measurements. Comput. Graph..

[36] Kim Y.-W., Joa K.-L., Jeong H.-Y., Lee S. (2021). Wearable IMU-based human activity recognition algorithm for clinical balance assessment using 1D-CNN and GRU ensemble model. Sensors.

[37] Mekruksavanich S., Phaphan W., Hnoohom N., Jitpattanakul A. (2024). Recognition of sports and daily activities through deep learning and convolutional block attention. PeerJ Comput. Sci..

[38] Müller P.N., Müller A.J., Achenbach P., Göbel S. (2024). IMU-based fitness activity recognition using CNNs for time series classification. Sensors.

[39] De Marchi M., Turetta C., Pravadelli G., Bombieri N. (2024). Combining 3-D human pose estimation and IMU sensors for human identification and tracking in multiperson environments. IEEE Sens. Lett..

[40] Liu L., Yang J., Lin Y., Zhang P., Zhang L. (2024). 3D human pose estimation with single image and inertial measurement unit (IMU) sequence. Pattern Recognit..

[41] Badawi A.A., Al-Kabbany A., Shaban H.A. (2020). Sensor type, axis, and position-based fusion and feature selection for multimodal human daily activity recognition in wearable body sensor networks. J. Healthc. Eng..

[42] Vidya B., Sasikumar P. (2022). Wearable multi-sensor data fusion approach for human activity recognition using machine learning algorithms. Sens. Actuators A Phys..

[43] Torabi S.M., Narimani M., Park E.J. (2025). In-home human activity recognition via kinematics-focused multimodal sensor fusion and spatio-temporal neural architecture. IEEE Sens. Lett..

[44] Liu K., Gao C., Li B., Liu W. (2024). Human activity recognition through deep learning: Leveraging unique and common feature fusion in wearable multi-sensor systems. Appl. Soft Comput..

[45] Xi Y., Huang T., Yang Z. (2026). Multimodal human action recognition and personalized sports health promotion: A deep learning framework integrating wearable sensor fusion. Front. Neurorobot..

[46] Zhang M., Huang Y., Liu R., Sato Y. (2025). Masked video and body-worn IMU autoencoder for egocentric action recognition. Computer Vision—ECCV 2024.

[47] Chen M., Tan G. (2024). 3D human pose estimation based on wearable IMUs and multiple camera views. Electronics.

[48] Daukantas S., Marozas V., Lukosevicius A., Jegelevicius D., Kybartas D. (2011). Video and inertial sensors based estimation of kinematical parameters in swimming sport. Proceedings of the 6th IEEE International Conference on Intelligent Data Acquisition and Advanced Computing Systems, Prague, Czech Republic, 15–17 September 2011.

[49] Lugaresi C., Tang J., Nash H., McClanahan C., Uboweja E., Hays M., Zhang F., Chang C.-L., Yong M.G., Lee J. (2019). MediaPipe: A framework for building perception pipelines. arXiv.

[50] Bazarevsky V., Grishchenko I., Raveendran K., Zhu T., Zhang F., Grundmann M. (2020). BlazePose: On-device real-time body pose tracking. arXiv.

[51] Zhang C.-L., Wu J., Li Y. (2022). ActionFormer: Localizing moments of actions with transformers. Computer Vision—ECCV 2022.

[52] Lin T.-Y., Goyal P., Girshick R., He K., Dollár P. Focal loss for dense object detection. Proceedings of the IEEE International Conference on Computer Vision (ICCV).

[53] Blackburn J., Ribeiro E. (2007). Human motion recognition using Isomap and dynamic time warping. Human Motion—Understanding, Modeling, Capture and Animation.

[54] Wijekoon A. (2019). MEx: Multi-Modal Exercise Dataset for Human Activity Recognition.

[55] Picerno P., Pecori R., Raviolo P., Ducange P. (2019). Smartphones and exergame controllers as BYOD solutions for the e-tivities of an online sport and exercise sciences university program. Higher Education Learning Methodologies and Technologies Online.

[56] Di Mitri D., Epp A., Schneider J. (2024). Preserving privacy in multimodal learning analytics with visual animation of kinematic data. Higher Education Learning Methodologies and Technologies Online.

